# Progress and indication for use of continuous glucose monitoring in patients with diabetes in pregnancy: a review

**DOI:** 10.3389/fendo.2023.1218602

**Published:** 2023-08-23

**Authors:** Yu Song, Xiaodan Zhai, Yu Bai, Cong Liu, Le Zhang

**Affiliations:** Department of Endocrinology, Shengjing Hospital of China Medical University, Shenyang, Liaoning, China

**Keywords:** gestational diabetes, continuous glucose monitoring, CGM, pregnancy outcome, perinatal outcome

## Abstract

Gestational diabetes mellitus is one of the most common endocrine diseases that occur during pregnancy. Disorders of blood glucose metabolism during pregnancy can increase the risk of adverse pregnancy outcomes, such as pregnancy-related hypertension, preeclampsia, eclampsia, miscarriage, macrosomia, and neonatal hypoglycemia. Continuous glucose monitoring (CGM) can safely and effectively monitor blood glucose changes in patients with gestational hyperglycemia, thereby reducing adverse pregnancy outcomes. Hence, this article aimed to provide a comprehensive review of the progress and indications for using CGM in pregnant patients with diabetes. CGM can reduce blood glucose fluctuations and the occurrence of serious hypoglycemia and hyperglycemia events and can provide time in range (TIR). TIR is an important indicator of blood glucose level. Patients with a higher TIR during pregnancy have better gestational outcomes.

## 1 Introduction

Diabetes is a common clinical complication of pregnancy, including gestational diabetes mellitus (GDM) and preexisting diabetes. Among these, GDM is the predominant type, accounting for 80–90% of pregnancies with hyperglycemia. According to the International Association of Diabetes and Pregnancy Study Groups (IADPSG), the global incidence of GDM is estimated to be 17.8% (1). Recent studies have shown that maternal pre-pregnancy body mass index is a potential modifiable risk factor for GDM. Moreover, this study showed that the incidence of GDM increased significantly with age. For women under 35 years of age, the prevalence of GDM is 16.4% in normal-weight, 23.0% in overweight, and 38.5% in obese women. For women over 35 years of age, the prevalence of GDM is 20.4%, 37.2%, and 51.4%, respectively (2).

With economic development and improvement in living standards, the prevalence of GDM has increased over the years (3), leading to increased adverse pregnancy outcomes in mothers and their offspring. For mothers, the incidences of dystocia, miscarriage, and eclampsia has increased (4). In the long term, the risk of type 2 diabetes mellitus (T2DM) in women with a history of GDM is nearly 10 times higher than that in women with normal blood glucose during pregnancy (5). The risks of macrosomia, neonatal hypoglycemia, hyperbilirubinemia, and neonatal respiratory distress syndrome are significantly increased in the offspring of women with GDM (4). A prospective study in 10–14-year-old children showed that the offspring of mothers with untreated GDM are at a high risk of impaired glucose tolerance (IGT). Among mothers with GDM, 10.6% of the children had IGT, whereas only 5.0% of the children of mothers without GDM had IGT. GDM is independently associated with children’s IGT (6). Therefore, monitoring and maintaining normal blood glucose levels during pregnancy is essential.

Currently, the commonly used clinical blood glucose monitoring methods include self-monitoring of blood glucose (SMBG), continuous glucose monitoring (CGM), hemoglobin A1c (HbA1c), and glycosylated albumin (GA). Many studies have recently shown that CGM is beneficial and widely used for the clinical treatment of patients with gestational diabetes. CGM can be real-time (rt-CGM) and intermittently scanned (is-CGM). It can continuously monitor glucose levels in subcutaneous tissue fluids and automatically record blood glucose levels at regular intervals to reflect blood glucose fluctuations accurately. CGM is employed for patients with diabetes during pregnancy, offering a more effective management approach in clinical settings. It enables clinicians to make better treatment selections and adjustments for patients, leading to optimal blood glucose control and improved pregnancy outcomes. This article reviews the use of CGM in pregnant women with diabetes.

## 2 Classification of pregnancy hyperglycemia

According to the American Diabetes Association (ADA) guidelines for 2023, pregnancy with hyperglycemia is categorized as GDM and preexisting diabetes (7).

GDM refers to a mild abnormality in glucose metabolism during pregnancy; however, the blood glucose level does not reach that of overt diabetes. During pregnancy, an increase in progesterone, cortisol, prolactin, and human placental hormone levels leads to the gradual aggravation of insulin resistance. Patients with GDM lack sufficient insulin production to combat the aggravation of insulin resistance, which leads to hyperglycemia. According to the diagnostic cut-off point established by IADPSG, GDM diagnostic criteria are: 75-g oral glucose tolerance test (OGTT) at any time during pregnancy, fasting blood glucose ≥ 5.1 mmol/L, 1-h OGTT blood glucose ≥ 10.0 mmol/L, and 2-h OGTT blood glucose ≥ 8.5 mmol/L. GDM can be diagnosed if one of the above mentioned blood glucose levels reaches the standard (8–10).

Pre-existing diabetes in pregnancy includes type 1 diabetes (T1DM), T2DM, or a special type of diabetes diagnosed before pregnancy, which is associated with the most severe hyperglycemia during pregnancy (8, 9). Pregnant women with T1DM have a higher risk of hypoglycemia and diabetic ketoacidosis than those with T2DM.The risk of hypertension and other comorbidities may be as high or higher in patients with T2DM than in those with T1DM (7).

## 3 Blood glucose monitoring of gestational diabetes

### 3.1 Hemoglobin A1c and glycosylated albumin

HbA1c reflects the average blood glucose level in the last 2–3 months (11). During pregnancy, red blood cell renewal is physiologically accelerated and the demand for iron increases exponentially (12), leading to a physiological decrease in HbA1c (13). In addition, increased vitamin C intake during pregnancy reduces HbA1c levels (14). Therefore, evaluating blood glucose control in patients with GDM using HbA1c levels is not accurate, as it can only serve as a supplementary reference for SMBG. Although several observational studies have shown that the level of HbA1c before pregnancy is associated with adverse pregnancy outcomes, such as fetal congenital malformation, premature delivery, preeclampsia, and perinatal death (15–17), the association between HbA1c level during the second trimester and adverse pregnancy outcomes has not been demonstrated (18, 19).

GA represents the blood glucose level within 2–3 weeks (20). An increase in GA levels can be observed in GDM (21), and GA can be used as a supplementary test for GDM diagnosis and blood glucose control monitoring (22). However, with increasing gestational age, GA continues to decrease, and the detection of GA has limited value in diagnosing gestational diabetes and predicting adverse pregnancy outcomes (23).

### 3.2 Self blood glucose monitoring

SMBG includes daily self-monitoring of fasting and postprandial blood glucose levels. The target values recommended by the ADA are as follows: fasting blood glucose < 5.3 mmol/L, 1-h postprandial blood glucose < 7.8 mmol/L, or 2-h postprandial blood glucose < 6.7 mmol/L (7). However, owing to multiple measurements of SMBG during pregnancy, long-term compliance is poor (24); hence, the fluctuation of blood glucose levels and the time spent within the target range cannot be readily displayed or interpreted. Errors often occur during clinical treatment processes, and new indicators are urgently needed.

### 3.3 Continuous glucose monitoring

CGM is an effective means of evaluating the fluctuation range of daytime and nighttime blood glucose levels in patients with diabetes. In the past decade, CGM has been proven to exhibit similar accuracy to that of SMBG (25) and can provide better treatment optimization, as well as indicate the trend of blood glucose, owing to its high test frequency (26). CGM can comprehensively analyze the patients’ blood glucose changes and provide information to patients and clinicians more intuitively by presenting an ambulatory glucose profile (AGP) and trend arrows. More importantly, CGM can also provide an alarm to help avoid serious hypoglycemic and hyperglycemic crises. CGM can improve the mental health and quality of life of patients by reducing the pain associated with fingertip blood sampling, thus improving compliance (27, 28). With its wide adoption in clinical practice, CGM can improve HbA1c and reduce glucose variability in patients with T1DM (29) and is more suitable for treatment monitoring than the use of SMBG in patients with T2DM (30). CGM is also widely used in patients with preexisting T1DM and T2DM during pregnancy and can improve gestational outcomes (31). Among women with GDM, CGM can provide a more comprehensive assessment of nocturnal hyperglycemia and improve the targeting of GDM interventions (32). CGM is also better than SMBG in detecting hypoglycemic episodes, which may improve maternal and fetal outcomes (26). Moreover, patient compliance is higher in CGM than in SMBG. In a prospective study, patient compliance in the CGM group was as high as 90%, which was significantly higher than that in the SMBG group (14). Therefore, CGM is recommended for patients with preexisting diabetes in pregnancy (especially T1DM complicated with pregnancy), GDM requiring insulin treatment, large blood glucose fluctuation, and potential nighttime hypoglycemia (33, 34).

In addition, a recent prospective cohort study of 73 women showed that CGM was well accepted among patients, could better demonstrate the blood glucose control of patients with GDM, and revealed the potential misdiagnosis of OGTT in GDM (35).Another pilot study conducted by the same team, involving 107 women, further validated the potential of CGM in detecting OGTT misdiagnosis. Additionally, CGM was more acceptable than OGTT to the participants (36).

## 4 Classification of CGM

### 4.1 Real-time continuous glucose monitoring

The rt-CGM system can provide a comprehensive glucose status for 3–14 days based on different needs. The device comprises a glucose-sensing device based on tiny glucose oxidase-filled electrodes and a glucose monitor connected by a cable. The system measures glucose concentration in the interstitial fluid every 5 min, continuously monitors glucose level for 24 h, and then forms an AGP. Rt-CGM has been extensively studied in patients with diabetes, and its clinical practicality has been demonstrated. It can detect postprandial hyperglycemia, nocturnal hyperglycemia, and hypoglycemia, which have not been previously reported. Rt-CGM displays not only glucose data in real time but also uses “arrows” to indicate the direction and rate of glucose changes, providing high and low blood glucose alarms and warnings. It can also provide data synchronization to enable timely intervention by the doctors and patients, thereby reducing the occurrence of serious hypoglycemia and hyperglycemia events (37–39). Moreover, CGM can improve the accuracy and effectiveness of clinical decision-making in patients with preexisting diabetes during pregnancy (40); however, the current rt-CGM system partially relies on SMBG for calibration.

### 4.2 Intermittently scanned continuous glucose monitoring

The current is-CGM system, also known as the instant glucose monitoring system, tracks glucose concentration in the human interstitial fluid approximately once every minute and requires scanning near the sensor placed on the skin to retrieve the data. Flash glucose monitoring is a typical example of is-CGM, which was identified by ADA in 2019 as a method that can replace SMBG for blood glucose monitoring (4). When the user scans the sensor, the current blood glucose value is recorded and retrospective reports for blood glucose data and related parameters, such as time in range (TIR), are generated (41). The is-CGM can be used for up to 14 days and does not need calibration with SMBG; however, it cannot deliver alerts (42).

Some studies have compared the two types of CGM and found that both is-CGM and rt-CGM can improve TIR, while rt-CGM has a greater percentage of TIR and can significantly reduce the incidence of hypoglycemia (43). When switching from is-CGM (FreeStyle Libre version 1) to rt-CGM (Dexcom G4) in 18 adult patients with T1DM, without changing insulin therapy management, there was an increase in TIR, a decrease in time below average (TBR), and no change in time above average (TAR) (44). Another study showed that in pregnant women with T1DM, no differences in TIR and TAR were observed, but women monitored by rt-CGM had a lower TBR compared to those monitored by is-CGM (45). Therefore, rt-CGM is more suitable for reducing the occurrence of hypoglycemia.

## 5 CGM indicators

In clinical practice, patients are recommended to wear CGM for 14 days. For patients with T1DM, 12–15 days of monitoring every 3 months can more accurately assess the level of blood glucose control (46, 47).

The CGM measurement value includes three key indicators: TIR (the proportion of time when the blood glucose is 3.9–10.0 mmol/L), TBR (proportion of time when blood glucose is <3.9 mmol/L), and TAR (proportion of time when blood glucose is>10.0 mmol/L). The main objective of effective and safe glucose control is to increase the TIR while reducing the TAR and TBR (48). Beck et al. found that in patients with diabetes mellitus, the probability of developing diabetic retinopathy and microalbuminuria increased by 64% and 40%, respectively, for every 10% reduction in TIR (49, 50). A study conducted among 141 pregnant women showed that among those with T2DM or GDM who utilized CGM, approximately 40% had TIR ≤ 70% and a higher likelihood of adverse neonatal and maternal outcomes compared to those with TIR > 70% (51). Murphy et al. pointed out that every 5% reduction in TIR and 5% increase in TAR in the second and third trimesters will increase the risk of being older than the gestational age, neonatal hypoglycemia, and admission to the neonatal intensive care unit (52). Therefore, it is necessary to improve the TIR levels in patients. In 2019, the TIR International Consensus recommended a TIR control target of >70% in pregnant women with T1DM. However, TIR control targets should be personalized. Patients with GDM and pregnant women with T2DM require more stringent targets and greater attention to overnight glucose (53).

In addition, common indicators of CGM include glucose management indicators, also called estimating A1C (54), blood glucose change rate [CV, target ≤ 36% (55)], and glycemic variability (GV). Patients with GDM risk factors have higher CV, and the corresponding incidence of adverse pregnancy outcomes is higher (56). GV in early pregnancy can be used as a potential predictor of subsequent GDM diagnosis. The mean amplitude of glycemic excursion, which is derived from GV, was significantly higher in patients with GDM (57).

## 6 CGM can better control blood glucose and improve pregnancy outcomes

Gestational diabetes increases the risk of pregnancy-related complications, such as hypertension, preeclampsia, eclampsia, premature rupture of membranes, cesarean section, postpartum hemorrhage, and intrauterine infection (58). Therefore, the management of blood glucose levels during pregnancy is very important for reducing adverse pregnancy outcomes. As shown in **Table 1**, many studies have reported that CGM can reduce adverse pregnancy outcomes. CGM provides patients with intuitive information on changes in blood glucose levels, enabling them to change their lifestyle and participate in treatment (59). Currently, CGM is being increasingly used in patients with gestational diabetes.

**Table 1:** the impact of CGM on pregnancy outcomes and perinatal outcomes.

Number	Country	Reference	Period	Size			Result		Recommendation
				TID	T2D	GDM	Maternal	Offspring	
1	UK, Austria	25	2018	24	11	39	The blood glucose measured by CGM and SMBG are highly consistent, and CGM reduces the pain and burden of users.	–	CGM is safe and accurate to use by pregnant women with diabetes.
2	Australia	32	2020	–	–	90	CGM data revealed nocturnal hyperglycemia in patients who were not commenced on insulin, with 60% of subjects breaching glucose targets overnight for >10% time. SMBG is hard to get such results.	–	CGM can make a more comprehensive assessment of nocturnal hyperglycemia.
3	Australia	35	2022	–	–	40	CGM can evaluate the diurnal pattern of glucose metabolism and has the potential to identify false positive and false negative OGTT.	–	CGM was well accepted and could better demonstrate the blood glucose control of GDM patients.
4	Sweden	45	2019	186	–	–	–	High maternal average blood glucose level and low TIR during pregnancy were associated with increased risk of LGA in offspring and comprehensive adverse outcomes in newborns.	Despite the use of CGM throughout pregnancy, daily blood glucose control is not ideal, and the incidence of LGA is still high.
5	Denmark	63	2021	20	–	–	The TBR measured by is-CGM is higher than that measured by rt-CGM.	–	The type of CGM device may affect the judgment of nocturnal hypoglycemia and thus affect the adjustment of nocturnal insulin dose.
6	England	52	2019	186	–	–	–	Every 5% reduction in TIR and 5% increase in TAR in the second and third trimesters will increase the risk of older than gestational age infants, neonatal hypoglycemia and admission to the neonatal intensive care unit.	Pregnant women should monitor TIR through CGM and raise the TIR to>70% as early as possible during pregnancy.
7	Australia	60	2007	8	10	37	CGM can show undetected postprandial hyperglycemia and overnight hypoglycemia.	–	CGM is a practical clinical tool with good compliance and is helpful for clinical decision-making.
8	England	62	2017	325	–	–	Pregnant CGM users spent more time in target and less time hyperglycemic, less hypoglycemia episodes and less time spent hypoglycemic.	Lower incidence of large for gestational age, fewer neonatal intensive care admissions lasting more than 24h, fewer incidences of neonatal hypoglycemia, and 1-day shorter length of hospital stay.	CGM should be provided to all pregnant women with type 1 diabetes who use intensive insulin therapy.
9	Holland	64	2018	109	82	109	CGM can reduce the incidence of gestational hypertension and preeclampsia in patients with type 1 diabetes and improve the level of HbA1c.	the use of is-CGM did not reduce the risk of macrosomia	CGM provides detailed information concerning glycemic fluctuations but, as a treatment strategy, does not translate into improved pregnancy outcome.
10	Worldwide	67	2022	–	–	482	Women with GDM using CGM may achieve lower average blood glucose levels and lower maternal weight gain.	Compared with using SMBG, patients using CGM to monitor blood glucose birth infants with lower birth weight	CGM is good for both mother and infant.
11	England	68	2021	–	–	100	the average blood glucose was more stable and TIR was higher in the group using is-CGM.	–	CGM may help to improve and treat the glucose tolerance disorder during pregnancy
12	China	69	2011-2012	–	–	340	Subjects in the CGM group were at lower risk of preeclampsia and primary cesarean delivery	The mean infant birth weight of women in the CGM group was lower	The use of supplementary CGM combined with routine antenatal care can improve the glycemic control and pregnancy outcomes of patients with GDM
13	Spain	74	2020	–	–	77	Every 1% increase in TAR would increase the probability of requiring drug treatment by 24%.	TAR was related to the occurrence of macrosomia and large for gestational age infants.	Using CGM to monitor the blood glucose changes of GDM patients can identify those patients who need drug treatment as early as possible, and reduce the occurrence of adverse pregnancy outcomes
14	England	76	2019	–	–	162	Mothers who delivered LGA infants had significantly higher blood glucose at night.	–	Using CGM to monitor and control the nocturnal blood glucose may help reduce the incidence rate of LGA in GDM women.
15	England	77	2008	46	25	–	–	Women in the CGM group delivered significantly smaller babies than the SMBG group	CGM during pregnancy is associated with improved glycemic control in the third trimester, lower birth weight, and reduced risk of macrosomia.

In a prospective study in Australia, 68 consecutive blood glucose monitoring examinations were conducted in 55 pregnant women. Sixty-two percent of the results provided important information for altering clinical management decisions, including postprandial and nocturnal hypoglycemia, and 77% of the participants acknowledged that CGM provided more benefits than inconvenience (60). CGM is a practical clinical tool with good compliance and is helpful in clinical decision-making.

The use of CGM is more suitable for the control of blood glucose levels, reduction of blood glucose fluctuations, and improvement of TIR in mothers with preexisting diabetes during pregnancy. Patients with T1DM have a high risk of developing severe hypoglycemia, which can have serious adverse effects on both the mother and fetus during pregnancy. Using CGM allows detection of glycemia fluctuations that might have gone unnoticed with intermittent blood glucose monitoring (61). An international study titled the CONCEPTT divided 325 women with T1DM into two groups. Only capillary blood glucose levels were monitored in one group, and CGM-assisted capillary blood glucose levels were monitored for the other group. Pregnant women who underwent CGM had a higher TIR and lower TAR and TBR. This report suggests that CGM should be administered to all pregnant women with T1DM receiving intensive insulin therapy (62). Viralnshah et al. conducted a prospective study and collected CGM data from 27 women with T1DM during pregnancy and found that TIR was significantly negatively correlated with HbA1c. For every 10% increase in TIR, HbA1c decreased by 0.3%, and the correlation between TIR and HbA1c in the second and third trimesters was stronger than that in the first trimester (r = -0.4) (63). Therefore, we assumed that CGM is suitable for pregnant women with T1DM, as it can help control blood glucose better.

A prospective study including 300 patients with gestational hyperglycemia found that CGM could reduce the incidence of gestational hypertension and preeclampsia in patients with T1DM and improve the level of HbA1c (64). However, although CGM can reduce the incidence of hypertensive disorders that complicate pregnancy in patients with diabetes, it does not significantly reduce the incidence of preeclampsia; the impact of CGM on preeclampsia remains to be discussed (65). Therefore, more robust evidence is required to confirm the effectiveness of CGM in improving pregnancy outcomes.

Although the blood glucose level in patients with GDM is much lower than that in patients with preexisting diabetes during pregnancy, its adverse effects on the future of the mother and fetus should not be underestimated. A follow-up study in Asia showed that women with a history of GDM had a high risk of developing T2DM in the future, and this risk increased with age (66).

García-Moreno et al. searched and screened a large number of studies and conducted a meta-analysis of 482 patients. Compared to women using traditional blood glucose monitoring methods, women with GDM using CGM may have lower average blood glucose levels, lower maternal weight gain, and lower birth weight of infants (67).

Majewska et al. recruited 100 women diagnosed with GDM and randomly assigned them to is-CGM and SMBG groups. The average blood glucose and total insulin resistance levels were determined. The average blood glucose was more stable and total insulin resistance was higher in the group using CGM, which may help to improve and treat glucose tolerance disorder during pregnancy (68).

One study found that the application of the CGM system can reduce the daily blood glucose fluctuation of patients with GDM by more than 25%, and the valley value of hyperglycemia can be significantly reduced (69, 70). This shows that CGM can better control blood glucose fluctuations and avoid excessive increases in blood glucose levels in patients with GDM. Compared to SMBG, CGM can reduce the average blood glucose level, increase the amplitude of maternal and infant birth weights, and improve pregnancy outcomes (68).

A randomized crossover study aimed to determine how the distribution of dietary carbohydrates affects blood glucose levels in women with GDM. CGM was used to monitor the blood glucose levels of 12 women with GDM undergoing diet treatment. The study concluded that “50% carbohydrate distribution in the morning is beneficial for reducing blood glucose and improving insulin sensitivity of women with GDM; however, it resulted in higher blood glucose variability.” Thus, women with GDM should reasonably manage their diet (71).

## 7 CGM improves perinatal outcomes

In patients with gestational diabetes, blood glucose level increases, leading to excessive glucose passing through the placenta and stimulating the pancreatic islets. This stimulation causes the fetus to produce excess insulin, resulting in increased synthesis of protein and fat in the fetus, consequently resulting in the development of a large baby (72). In addition, owing to excessive insulin production, hypoglycemia can occur easily when the fetus separates from the mother during childbirth. If glucose is not supplemented in time, the incidence of hypoglycemia increases. Both hyperglycemia and hyperinsulinemia can reduce the surface-active substance of fetal lung type II cells, hindering the growth of the fetal lung and affecting its normal development. This condition can lead to neonatal respiratory distress syndrome (73). Poor blood glucose control during pregnancy can result in adverse perinatal outcomes. As shown in **Table 1**, several studies have reported that CGM reduces adverse perinatal outcomes.

In a prospective study, CGM was used to monitor blood glucose changes in 77 patients with GDM at 26–32 weeks of gestation for 6days. The pattern of hyperglycemia before, after, and at night and its correlation with maternal and fetal complications and drug treatment were analyzed. TAR was related to the occurrence of macrosomia and large-for-gestational-age (LGA) infants. Every 1% increase in TAR increased the probability of requiring drug treatment by 24%. Using CGM to monitor blood glucose changes in patients with GDM enables identification of patients who require drug treatment at an early stage. This proactive approach can help reduce the incidence of adverse pregnancy outcomes, such as macrosomia (74).

LGA infants are referred as newborns whose birth weight is above the 90th percentile of the average weight of infants at the same gestational age, which is closely related to the increase in maternal blood glucose. Long-term glucose metabolic dysfunction may increase the risk of macrosomia (75). A prospective observational study was conducted using CGM in 162 pregnant women with GDM for 7 days at 30–32 weeks of gestation. Using the blood glucose index and blood glucose variability measurements provided by CGM, functional data analysis showed that mothers who delivered LGA infants had significantly higher blood glucose levels at night. Monitoring and controlling nocturnal blood glucose levels may help further reduce the incidence rate of LGA infants in women with GDM (76).

The CONCEPTT study pointed out that compared with SMBG, patients who underwent CGM had significantly improved newborn health outcomes, including a reduced incidence of LGA infants, fewer neonatal intensive care inpatients lasting more than 24 h, a decreased occurrence of neonatal hypoglycemia, and a shortened hospitalization period by one day (62). The use of CGM during pregnancy in patients with T1DM is related to an improvement in neonatal outcomes, which may be attributed to a reduction in maternal hyperglycemia exposure.

Murphy et al. studied the effects of CGM on the offspring of pregnant women with T1DM (46 women) or T2DM (25 women). These women were randomly assigned to the CGM and standard prenatal treatment group (CGM+SMBG, 38 women) or the standard prenatal treatment group (SMBG, 33 women). Women in the CGM group, as measured by the median percentile of birth weight, eventually delivered significantly smaller babies than those in the SMBG group. However, no significant difference was observed between the two groups in terms of LGA infants, cesarean section, preeclampsia, or other indicators used to measure the incidence rate of newborns (77).

Similarly, Kristensen et al. conducted a prospective study of 186 pregnant women with T1DM in Sweden, 92 of whom underwent rt-CGM and 94 underwent is-CGM. The number of LGA infants was similar in rt-CGM and is-CGM users, and high maternal average blood glucose levels and low TIR during pregnancy were associated with an increased risk of LGA and comprehensive adverse outcomes in newborns. However, the rt-CGM group exhibited a lower TBR than the is-CGM group. Therefore, although the impact of rt-CGM on perinatal outcomes was not significantly different from that of is-CGM, rt-CGM was still more suitable for reducing the occurrence of hypoglycemia (45). However, another study showed that intermittent rt-CGM use during pregnancy did not improve blood glucose control or pregnancy outcomes in women with GDM (76).

In summary, there are still few controversial findings regarding CGM improving perinatal outcomes in patients with gestational diabetes. Therefore, a large number of prospective studies are needed to explore the effectiveness of CGM in improving perinatal outcomes in patients with gestational diabetes.

## 8 Summary

The prevalence of gestational diabetes is increasing with improvements in living standards. Blood glucose monitoring is the basis for GDM management. The goal of GDM treatment is to minimize maternal and fetal adverse events related to hyperglycemia or severe hypoglycemia. Several clinical studies have demonstrated that satisfactory glucose control during pregnancy effectively reduces maternal and infant complications. CGM can effectively monitor blood glucose changes in patients with diabetes during pregnancy, thereby providing better guidance for clinical treatment. Therefore, CGM is recommended for patients with preexisting diabetes in pregnancy (especially T1DM complicated with pregnancy), GDM requiring insulin treatment, large blood glucose fluctuations, and possible nighttime hypoglycemia. This article reviews the use of CGM in patients with diabetes during pregnancy, and many studies have confirmed that CGM can improve pregnancy outcomes. However, there is still some controversy about the impact of CGM on maternal and infant health, which necessitates further discussion and clarification using big data and large samples.

## Author contributions 

YS wrote the first draft of the manuscript and edited it. XZ summarized the manuscript and drew the **Table 1**. YB and CL reviewed literature and organized them. LZ performed critical revision of the literature and editing of the manuscript. All authors contributed to the article and approved the submitted version.
